# Correlation between red blood cell distribution width-to-albumin ratio and diabetic retinopathy

**DOI:** 10.6026/973206300211324

**Published:** 2025-06-30

**Authors:** Prathibha Elavala, Chaitra M.C, Prabhavathi K., Suresh T.N

**Affiliations:** 1 Department of Ophthalmology, Sri Devaraj Urs Medical College, Tamaka, Kolar, Karnataka, India; 2 Department of Biochemistry, Sri Devaraj Urs Medical College, Tamaka, Kolar, Karnataka, India; 3 Department of Pathology, Sri Devaraj Urs Medical College, Tamaka, Kolar, Karnataka, India

**Keywords:** Diabetes mellitus, diabetic retinopathy, red blood cell distribution width (RDW), albumin ratio, prognosis

## Abstract

Diabetic retinopathy is always asymptomatic before entering the later stage. Therefore, it is of interest to determine the red cell distribution width to albumin ratio (RAR) and correlate RAR with Diabetic Retinopathy through cross-sectional study among 384 patients. On applying Z test, there was a statistically significant difference found between RAR and Presence of diabetic retinopathy. These findings suggest that RAR could serve as a useful biomarker for early detection and risk stratification of DR.

## Background:

Diabetes mellitus (DM) has shown a trend of reaching pandemic levels in the world. Diabetic retinopathy (DR) is a microvascular complication of DM and is a primary cause of acquired blindness among working-age individuals [1]. Diabetic retinopathy is always asymptomatic before entering the later stage because diabetic retinopathy seriously harms human health and the economic sustainability of the national health system, both for individual vision and society [2]. Various mechanisms and factors mediate diabetic retinopathy development, including pregnancy, diabetic nephropathy, obesity, family history, blood glucose fluctuations, hyperlipidemia, chronic diabetes, hypertension and hyperglycemia. The pathogenesis of diabetic retinopathy is not fully understood; however, several studies suggested that inflammation plays an important role [3]. Chronic inflammation may contribute to the development of macroangiopathy and macroangiopathy in patients with diabetes. Red blood cell distribution width (RDW) is a parameter that reflects the heterogeneity of red blood cells (RBC). Elevated red blood cell distribution width is associated with severe imbalance of RBC homeostasis, abnormal RBC production and survival. This may be associated with oxidative stress, vascular inflammation, and malnutrition such as iron, vitamin B12, and folic acid deficiencies. In addition, excessive red blood cell distribution width may increase mortality from diabetes mellitus and macrovascular as well as macrovascular diseases [4]. Serum albumin plays critical physiological roles, including maintaining oncotic pressure, transporting various substances such as hormones, fatty acids and contributing to antioxidant deference, making it a multifunctional protein with significant implications for overall health. Recent studies have shown that albumin is involved in inflammation [5, 6]. The red cell distribution width-to-Albumin ratio (RAR) is a comprehensive and novel biomarker combining inflammation & nutritional status that reflects patient's frailty. The role of RAR in patients with diabetic retinopathy is still unclear with only one study by Zhao *et al.* [4]. Therefore, it is of interest to assess the association between diabetic retinopathy and RDW-to Albumin levels to determine the value of RAR in predicting DR.

## Methodology:

## Study design:

A cross-sectional study

## Study duration:

6 months

## Study population:

All patients > 18 years with type 2 Diabetes mellitus attending Out Patient Department of Ophthalmology at R. L. Jalappa Hospital and Research, Kolar which is attached to Sri Devaraj Urs Medical College.

## Sample size:

384 patients of type 2 Diabetes mellitus

## Inclusion criteria:

Type II Diabetic mellitus patients aged > 18 years.

## Exclusion criteria:

[1] Patients with coronary Heart disease, Hypertension & chronic Kidney disease, Chronic liver disease,

[2] Systemic infectious diseases,

[3] Hematologic diseases,

[4] Media opacity obscuring the visualization of fundus. (mature cataract, corneal opacity, corneal ulcer)

[5] Uveitis patients

[6] Malnutrition

After obtaining Ethical clearance from the Institutional ethics committee, a written informed consent from the subjects was obtained prior to the start of study. Detail demographic information including age, gender, address, socio-economic status and medical history including duration of disease, diabetic complications and diabetic history in family was collected from the subjects. After a detailed history, following clinical examination was performed:

[1] Visual acuity by Snellen's chart for distant vision.

[2] Near vision by Jaeger's chart.

[3] Autorefractometry

[4] Slit lamp biomicroscope for Anterior segment evaluation

[5] Intraocular Pressure by Goldmann applanation tonometry

[6] Fundus examination by slit lamp biomicroscope using 90D lens, Direct & Indirect ophthalmoscopy.

Based on the findings of the fundus, patient will be classified as Diabetic Retinopathy (DR) or No Diabetic Retinopathy.

Further diabetic retinopathy is classified based on Early Treatment Diabetic Retinopathy Study (ETDRS) Classification as follows:

A) Non-proliferative diabetic retinopathy:

1) No retinopathy: No retinal lesions

2) Very mild NPDR: Microaneurysms only

3) Mild NPDR: A few microaneurysms, retinal haemorrhages & hard exudates

4) Moderate NPDR: Retinal haemorrhages (about 20 medium-large per quadrant) in 1-3 quadrant + cotton wool spots (between the grades mild and severe NPDR)

5) Severe NPDR: fulfilling one rule of the 4-2-1 rule.

4-2-1 rule:

a) Severe haemorrhages in all four quadrants

b) Venous beading in 2 or more quadrants

c) Moderate IRMA in 1 or more quadrants

6) Very Severe NPDR: fulfilling two or more rules of the 4-2-1 rule [36-37].

B) Proliferative diabetic retinopathy:

1) Mild to moderate PDR- NVD or NVE insufficient to meet high-risk characteristics

2) High-risk PDR:

a) NVD greater than ETDRS standard photograph 10A (about 1/3 disc area).

b) Any NVD with vitreous haemorrhage.

c) NVE greater than 1/2 disc area with vitreous haemorrhage.

C) Advanced Diabetic Eye Disease is the end-stage vision-threatening complication of diabetic retinopathy in patients whose treatment is inadequate or unsuccessful. It may present as pre-retinal or intraretinal haemorrhage, tractional retinal detachment, or rubeosis iridis.

## Operational definition of DM:

The selection criteria for diabetes included self-reported diabetes, on anti-diabetes drugs, taking insulin, fasting glucose ≥110 mg/dl, or glycohemoglobin (HbA1c) ≥6.5%. As per the above definition, all selected Diabetes mellitus patients, after obtaining a written & informed consent, demographic characteristics were noted which includes age, sex, race, marital status, body mass index (BMI), C-reactive protein (CRP), RDW, Albumin, Insulin use & fasting glucose. After a detailed history, following clinical examination was performed:

[1] Visual acuity by Snellen's chart for distant vision.

[2] Near vision by Jaeger's chart.

[3] Autorefractometry

Red cell distribution width in percentage was measured by SYSMEX XN1000 analyzer using 2ml peripheral blood collected in Ethylene Diamine Tetra-acetic Acid (EDTA) vacutainer and the serum Albumin concentration was determined using 3 ml blood by Bromocresol green method in VITROS5600 analyzer. Then using the red blood cell distribution width (%) /Albumin (g/dl) = (RAR) was determined.

## Statistical analysis:

Data was entered into Microsoft excel data sheet and was analyzed using SPSS 22 version software. Categorical data was represented in the form of Frequencies and proportions. Chi-square test or Fischer's exact test (for 2x2 tables only) was used as test of significance for qualitative data. Continuous data was represented as mean and standard deviation. Independent t test was used as test of significance to identify the mean difference between two quantitative variables. P value (Probability that the result is true) of <0.05 was considered as statistically significant after assuming all the rules of statistical tests.

## Results and Discussion:

The mean age among presence of diabetic retinopathy was 63.96 years and among non-diabetic retinopathy group was 62.17 years. On applying Z test, there was a statistically significant difference found between mean age and Presence of diabetic retinopathy (Table 1 - see PDF & Figure 1 - see PDF). Out of total in diabetic retinopathy group 110(64%) cases were males and remaining 62(365) cases were females. Similar results were found in other group also. On applying chi square test, there was no association found between gender and occurrence of diabetic retinopathy (Table 2 - see PDF & Figure 2 - see PDF). On applying Z test, there was a statistically significant difference found between Duration of diabetes mellitus, BMI as well as CRP and Presence of diabetic retinopathy (Table 3 - see PDF). On applying Z test, there was no statistically significant difference found between RDW, Albumin and Presence of diabetic retinopathy. On applying Z test, there was a statistically significant difference found between RAR and Presence of diabetic retinopathy (Table 4 - see PDF).

The findings of this study highlight the significant correlation between the red blood cell distribution width-to-albumin ratio (RAR) and diabetic retinopathy (DR), supporting the role of inflammation and nutritional status in the pathogenesis of DR. The mean age of patients with diabetic retinopathy was significantly higher than those without DR, aligning with previous studies that suggest aging as a contributing factor to retinal microvascular damage [8]. Additionally, male predominance in diabetic retinopathy cases was noted, consistent with findings by Zhao *et al.* [9], who reported a higher prevalence of diabetic retinopathy among males, possibly due to differences in metabolic and hormonal factors. The duration of diabetes was significantly longer in the diabetic retinopathy group, corroborating evidence from multiple studies that demonstrate a strong association between prolonged hyperglycemia and retinal damage [10]. Body mass index (BMI) was also found to be significantly higher in diabetic retinopathy patients, in agreement with previous research suggesting that obesity exacerbates insulin resistance and vascular inflammation, increasing diabetic retinopathy risk [11]. Inflammation is a critical contributor to diabetic retinopathy progression, as indicated by elevated C-reactive protein (CRP) levels in diabetic retinopathy patients. Elevated CRP has been linked to endothelial dysfunction and increased vascular permeability, promoting diabetic retinopathy development. Although the study did not find a significant difference in red blood cell distribution width or albumin levels between diabetic retinopathy and non-DR groups, the significant elevation of RAR in diabetic retinopathy patients suggests that this novel biomarker may serve as a more sensitive predictor of diabetic retinopathy progression compared to its individual components. Zhao *et al.* [7] similarly found that higher RAR levels were associated with advanced stages of DR, further supporting its clinical utility. The clinical implications of this study are noteworthy. Given that RAR integrates markers of inflammation and nutritional status, it may serve as an accessible and cost-effective biomarker for diabetic retinopathy screening, especially in resource-limited settings. Further prospective studies with larger sample sizes are necessary to validate RAR as a predictive tool and explore its potential role in guiding therapeutic interventions for DR.

## Prior publication:

Not Sent.

## Support:

Nil

## Permissions:

Nil

## Conclusion:

A significant association between RAR and the presence of diabetic retinopathy in patients with type II diabetes mellitus is shown, reinforcing the role of inflammation and nutritional status in its pathogenesis. These findings suggest that RAR could serve as a useful biomarker for early detection and risk stratification of DR, ultimately aiding in better clinical management and prevention strategies.
